# Functional and Pharmacological Characteristics of Permeability Transition in Isolated Human Heart Mitochondria

**DOI:** 10.1371/journal.pone.0067747

**Published:** 2013-06-28

**Authors:** Saori Morota, Theodor Manolopoulos, Atli Eyjolfsson, Per-Ola Kimblad, Per Wierup, Carsten Metzsch, Sten Blomquist, Magnus J. Hansson

**Affiliations:** 1 Mitochondrial Pathophysiology Unit, Skåne University Hospital & Lund University, Lund, Sweden; 2 Department of Cardiothoracic Anesthesiology and Intensive Care, Skåne University Hospital & Lund University, Lund, Sweden; 3 Department of Cardiothoracic Surgery, Skåne University Hospital & Lund University, Lund, Sweden; 4 Department of Clinical Physiology, Skåne University Hospital & Lund University, Lund, Sweden; University of Mississippi, United States of America

## Abstract

The objective of the present study was to validate the presence and explore the characteristics of mitochondrial permeability transition (mPT) in isolated mitochondria from human heart tissue in order to investigate if previous findings in animal models of cardiac disorders are translatable to human disease. Mitochondria were rapidly isolated from fresh atrial tissue samples obtained from 14 patients undergoing Maze surgery due to atrial fibrillation. Human heart mitochondria exhibited typical mPT characteristics upon calcium overload such as swelling, evaluated by changes in light scattering, inhibition of respiration and loss of respiratory coupling. Swelling was a morphologically reversible event following transient calcium challenge. Calcium retention capacity (CRC), a quantitative measure of mPT sensitivity assayed by following extramitochondrial [Ca^2+^] and changes in respiration during a continuous calcium infusion, was significantly increased by cyclophilin D (CypD) inhibitors. The thiol-reactive oxidant phenylarsine oxide sensitized mitochondria to calcium-induced mPT. Release of the pro-apoptotic intermembrane protein cytochrome *c* was increased after, but not before, calcium discharge and respiratory inhibition in the CRC assay. From the present study, we conclude that adult viable heart mitochondria have a CypD- and oxidant-regulated mPT. The findings support that inhibition of mPT may be a relevant pharmacological target in human cardiac disease and may underlie the beneficial effect of cyclosporin A in reperfusion injury.

## Introduction

The mitochondrial permeability transition (mPT) is considered to be a major cause of cell death in ischemia-reperfusion injury of the heart. Opening of the mPT pore is characterized by uncoupling of oxidative phosphorylation, in vitro swelling of mitochondria and release of proapoptotic factors such as cytochrome *c* (CytC) [1], [2]. Pharmacological inhibition or genetic ablation of the mitochondrial matrix protein cyclophilin D (CypD) prevents mPT and cardiac injury in animal models of ischemia-reperfusion injury and heart failure [3]–[7]. Ischemic preconditioning has been proposed to exert its beneficial effect through reduced mPT activation, although the signaling pathways remain to be fully elucidated [8]–[11]. The immunosuppressive agent and CypD inhibitor cyclosporin A (CsA) has also been shown to limit myocardial injury in a Phase II clinical trial of patients with acute myocardial infarction [12], [13]. CsA and other cyclophilin inhibitors are however not specific to CypD. Cyclophilins are found widely distributed in eukaryotes in all the major compartments of the cell, and the majority of the 17 identified human cyclophilins have cytoplasmic or nuclear localization [14]. The complex of cytoplasmic cyclophilin A and CsA inhibits the phosphatase calcineurin, which mediates the immunosuppressive activity of CsA [15].

An important step in translating experimental findings to clinical use and to increase the strength of the biologic rationale for treatment is to verify the pharmacological target in human tissue. Previously, mPT has been implicated indirectly in human atrial heart tissue by demonstrations of improved atrial trabeculae and myocyte viability following simulated ischemia in vitro and by prolonged time to depolarization following tetramethylrhodamine methyl ester (TMRM)-induced oxidative stress by cyclophilin inhibitors [16], [17]. Repetitive calcium loads has also been shown to cause respiratory inhibition in permeabilized human atrial myofibres [18]. Even though cellular assays posses several strengths, the specificity may be lower compared to studies in isolated mitochondria with increased risk of confounding variables both in regard to the studied phenomena and the pharmacological effects. There is no previous study exploring the specific characteristics of permeability transition or the direct effect and potencies of cyclophilin inhibitors in isolated human heart mitochondria.

The objective of the present study was to confirm the presence of mPT in the human heart by assessing characteristics of mPT in freshly isolated human heart mitochondria. Further, the aim was to explore the pharmacological modulation of mPT by CypD inhibitors in order to evaluate whether mPT constitutes a relevant target for cardioprotection in pathologies of the heart where this disease mechanism has been implicated in animal models. The study demonstrates that viable mitochondria from human cardiac tissue undergo calcium- and oxidant-sensitive mPT similar to what has previously been described in non-human mitochondria and human brain and liver mitochondria [19], [20], and that its activation is dose-dependently inhibited by CypD ligands.

## Materials and Methods

### Material

To obtain fresh human heart tissue for functional mitochondrial analyses, left atrial appendage tissue samples were collected from 14 patients undergoing Maze surgery due to atrial fibrillation at the Skåne University Hospital, Lund, Sweden. For further patient characteristics, see Table 1. In Maze surgery, incisions are performed in the atria to disrupt abnormal electrical impulses and the left atrial appendage is removed. Tissue samples which would otherwise have been discarded, 0.3–4.3 g, were transferred into ice-cold Buffer A (100 mM KCl, 50 mM MOPS, 5 mM MgCl_2_, 1 mM EGTA, 1 mM ATP(K), pH 7.4).

**Table 1:** Patient characteristics.

Age, median (range)		71 (55–81) years
Sex	Male	11 (79%)
	Female	3 (21%)
Previous AMI^a^		6 (43%)
Diabetes mellitus		3 (21%)
Medication	Nitroglycerin	1 (7%)
	ACE^b^ inhibitors	10 (71%)
	Aspirin	5 (36%)
	Beta-blocker	10 (71%)
	Statin	12 (86%)
	Calcium channel blocker	6 (43%)
	Digoxin	2 (14%)

a AMI = acute myocardial infarction, ^b^ACE = Angiotensin-converting enzyme.

### Ethics Statement

The study procedures were approved by the regional ethical review board of Lund, Sweden (permit number 2009/507) and comply with the World Medical Association Declaration of Helsinki - Ethical Principles for Medical Research Involving Human Subjects. Samples were obtained after written informed consent was acquired.

### Isolation of Heart Mitochondria

Heart tissue samples were rapidly prepared for mitochondrial isolation. Non-muscle tissue was removed and remaining muscle was finely chopped in ice-cold Buffer A with BSA (2 mg/ml) [21], [22]. After rinsing off BSA by adding excess ice-cold Buffer A, tissues were transferred to a Potter-Elvehjem homogenizer and trypsinized (10 mg trypsin/6 ml ice-cold Buffer A) for 30 minutes on ice. BSA, 12 mg, was added to stop the trypsinization, and the tissue was homogenized gently with a Teflon pestle. Following 10 minutes centrifugation at 600 *g*, supernatant was collected and centrifuged for 5 minutes at 3000 *g*. The pellet was suspended in 8 ml of 26% Percoll solution in Buffer B (100 mM KCl, 50 mM MOPS, 0.5 mM EGTA, pH 7.4) and centrifuged for 7 minutes at 30000 *g* to remove contaminating membranes [23]. The pellet was resuspended in 8 ml of Buffer B with BSA (0.2 mg/ml Buffer B) and centrifuged for 3 minutes at 7000 *g* to wash away Percoll. A second washing step in Buffer B without BSA was performed with centrifugation for 3 minutes at 3000 *g*. The mitochondrial pellet was finally resuspended in Buffer B. Protein content was measured using Bradford analysis after which 1 mg/ml BSA was added. All centrifugations were performed at 4°C.

### Mitochondrial Respiration

Oxygen consumption of mitochondria was analyzed using an Oxygraph-2k with a Titration-Injection microPump TIP-2k (Oroboros instruments, Innsbruck Austria). Experiments were performed at 37°C. Mitochondria, 40 µg, were suspended in 2 ml respiration medium (MIR05) containing 110 mM sucrose, 20 mM HEPES, 20 mM taurine, 60 mM K -lactobionate, 3 mM MgCl_2_, 10 mM KH_2_PO_4_, 0.5 mM EGTA, 1 g/l BSA and 5 mM of the NADH-linked respiratory substrates malate and glutamate, pH 7.1. Mitochondrial suspensions were supplemented with 0.25 mM ADP to induce state 3 respiration and to evaluate respiratory coupling of the isolated mitochondrial preparations. State 4 respiration was measured after the ADP was consumed. In another experimental group, mitochondria were exposed to 1 mM CaCl_2_ to evaluate the effect of calcium-induced mPT on respiratory function. Then, 1 mM ADP was added in both experimental groups followed by the ATP synthase inhibitor oligomycin, 1 µg/ml, to induce State 4_oligo_. A stepwise titration of the protonophore CCCP (500 nM/addition) was performed to evaluate maximal capacity of the electron transport system (ETS) independent of the phosphorylation system.

### De-energized Swelling

A Perkin-Elmer Luminescence Spectrometer LS-50B (Emeryville, CA, USA) with a temperature controlled cuvette holder was used for all fluorescence and light scattering experiments. De-energized swelling experiments were performed at 28°C in a 150 mM KCl-based buffer containing 0.5 µM rotenone, 0.2 mg/ml antimycin A, 2 µM calcium ionophore A23187, 0.5 mM PPi. Mitochondria were pre-treated for two minutes with 10, 100, 1000 nM CsA or 10, 100, 1000 nM of the non-immunosuppressive cyclosporin analog MeAla^3^EtVal^4^-cyclosporin (NI-Cs, also known as alisporivir, UNIL025, Debio-025 or DEB025). The mitochondrial suspensions were then exposed to 300 µM CaCl_2_ to induce swelling. The extent of swelling was calculated by dividing the calcium-induced decrease in light scattering during the first minute following CaCl_2_ addition with that induced by the ionophore alamethicin (10 µg/ml) [24].

### Reversible Swelling

The reversibility of calcium-induced swelling was evaluated in respiring mitochondria at 37°C. Experiments were performed in buffer containing 125 mM KCl, 20 mM Trizma base, 2 mM Pi (K), 1 mM MgCl_2_, 1 µM EGTA and 5 mM of the NADH-linked respiratory substrates malate and glutamate, pH 7.1. Following 2 minutes exposure of mitochondria to 300 µM CaCl_2_ to induce swelling, 0.5 mM EGTA was added to chelate the CaCl_2_. A second exposure of CaCl_2_, using 400 µM, was performed following 11 minutes of recovery. Experiments were terminated by adding 10 µg/ml alamethicin, and carried out with or without 1 µM CsA.

### Calcium Retention Capacity (CRC)

Mitochondrial CRC was evaluated using both measurements of calcium fluxes and changes in respiration during a continuous CaCl_2_ infusion using the luminescence spectrometer and oxygraph described above. Mitochondrial Ca^2+^ uptake and release were monitored by following the excitation ratio of the extramitochondrial calcium-sensitive fluorescent probe Fura 6F (250 nM, Ex. 340/380 nm, Em. 509 nm). Release of sequestered calcium or initiation of respiratory inhibition were attributed to activation of mPT [25]. Mitochondria, 40 µg, were suspended in 2 ml buffer containing 125 mM KCl, 20 mM Trizma base, 2 mM Pi (K), 1 mM MgCl_2_, 1 µM EGTA, 200 µM ATP, 10 µM BSA, 5 mM of malate and glutamate, pH 7.1. At start of experiment, 1 µg/ml oligomycin, 50 µM ADP and then 1 µM CsA, 1 µM NI-Cs, 1 µM of the vicinal thiol reagent phenylarsine oxide (PhArs) or vehicle (ethanol) was added. The suspensions were infused with 0.2 µmol CaCl_2_•min^–1^•mg^–1^. CRC was calculated as the amount of infused calcium from the start of infusion until start of maximal calcium release or start of the rapid phase of respiratory inhibition.

### Cytochrome *C* Release

The extent of cytochrome *c* (CytC) release was evaluated in the CRC experimental setup described above. Samples were prepared before and during calcium infusion as well as after induction of respiratory inhibition. Samples were also collected following incubation of mitochondria with 10 µg/ml alamethicin. An ELISA kit for detection of human CytC (Quantikine®, R&D Systems) was employed to measure the extent of its release as described previously [26].

### Electron Micrographs

Mitochondrial morphology was evaluated in the CRC and reversible swelling experimental setups as described above. Samples were prepared before or during calcium infusion and after induction of respiratory inhibition in the CRC assay. In the reversible swelling experiments, samples were collected before and after the first CaCl_2_ addition, following the light scattering recovery after EGTA chelation of CaCl_2_, after second CaCl_2_ exposure and following addition of 10 µg/ml alamethicin. The suspensions were rapidly chilled and centrifuged in an Eppendorf microcentrifuge, 12000 *g*, for 2 minutes. Samples were fixed in a solution containing 0.1 M Sörensen buffer, 1.5% Paraformaldehyde and 1.5% Glutaraldehyde over night, and further processed as described previously [27].

### Statistical Analyses

All average results are presented as mean ± SD and were, unless otherwise noted, evaluated using student’s t-tests or for multiple groups one-way ANOVA followed by Dunnett’s multiple comparison post hoc test using GraphPad Prism v5.0 software. Differences were considered significant where *P*<0.05.

## Results

### Functional Integrity of Isolated Mitochondria

**Figure 1:**
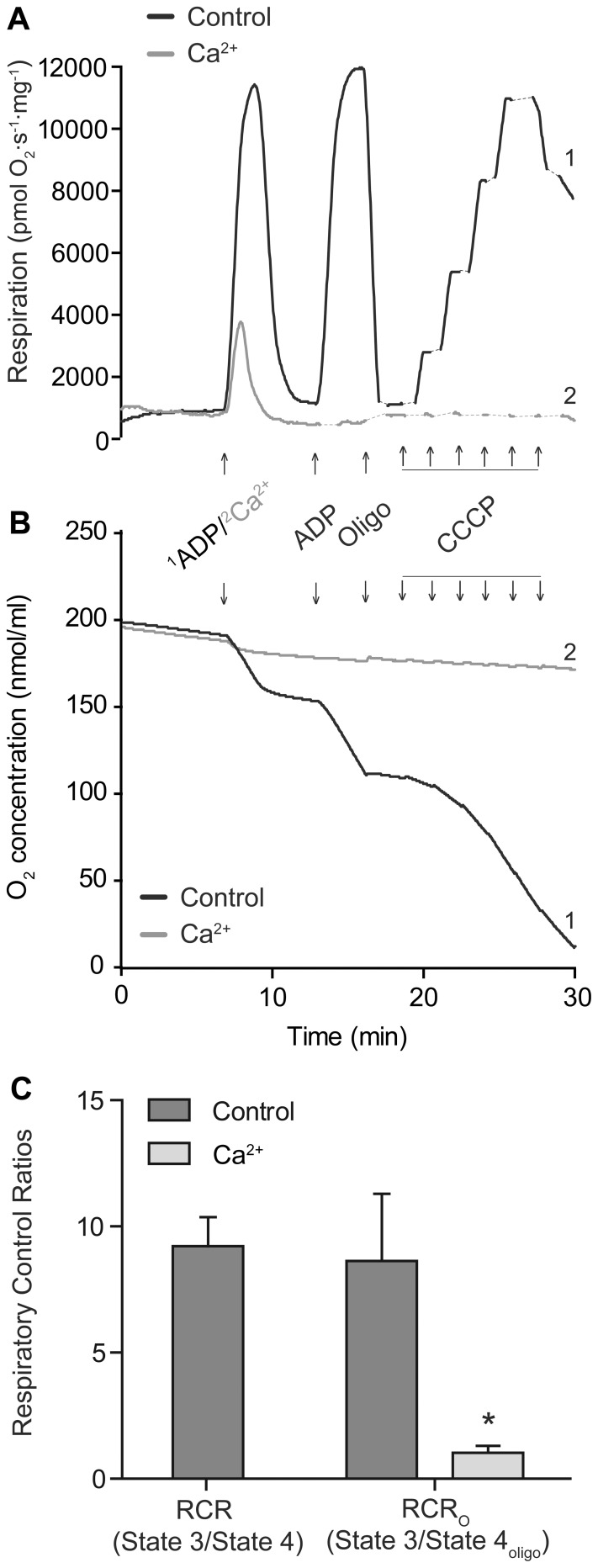
Respiration of isolated heart mitochondria with and without exposure to calcium. **A)** Representative traces of changes in respiration rates and **B)** decrease of oxygen concentration in the closed chambers. In black traces (1), 0.25 mM ADP was added to induce State 3 respiration. After the initial ADP was consumed (State 4 respiration) a second addition of ADP, 1 mM, was added followed by the ATP synthase inhibitor oligomycin (Oligo) to induce State 4_oligo_ respiration. Finally, the protonophore CCCP was titrated, 0.5 µM per addition, to induce maximal non-phosphorylation-dependent respiration. In gray traces (2), the initial ADP addition was replaced by 1 mM CaCl_2_. **C)** Calculated respiratory control ratios (RCR) of mitochondria with or without exposure to CaCl_2_. RCR (State 3/State 4) is depicted for control mitochondria and RCR_O_ (State 3/State 4_oligo_) for both experimental groups. Values are means ± SD, n = 4. *indicate p<0.05 using student’s t-test.

The mitochondria isolated from fresh atrial heart tissue (isolation yield 376.1±205.2 µg mitochondria/g tissue) displayed good coupling of oxidation to ATP production. The state 3 and state 4 respiratory rates were 9157.1±2430.4 and 994.9±249.3 pmol O_2_•s^–1^•mg^–1^, respectively, with a respiratory control ratio (RCR) of 9.22±1.15 (Fig. 1). State 4 respiration following addition of the ATP synthase inhibitor oligomycin (State 4_oligo_) was not different from state 4 respiration without oligomycin (1054.5±54.5 pmol O_2_•s^–1^•mg^–1^, p = 0.66), indicating that there was no contaminating ATPase activity, *e.g.* disrupted mitochondria, in the preparations.

### Calcium–induced Alterations of Mitochondrial Respiration

The coupling of oxidative phosphorylation was virtually lost in mitochondria following a calcium exposure. There was no respiratory stimulation upon ADP addition and the RCR_O_ (State 3/State 4_oligo_) decreased from 8.64±2.65 in control samples to 1.13±0.28 in mitochondria exposed to calcium (Fig. 1). A permeable inner membrane in mitochondria oxidizing complex 1-linked substrates will besides loss of proton motive force also lead to respiratory inhibition due to dilution of the NAD(H) pool [25], [26], and a disrupted outer membrane following mitochondrial swelling may cause respiratory inhibition due to loss of CytC [28]. Upon calcium addition, there was a transient increase in respiration followed by a decrease in all respiratory rates. Titration of the protonophore CCCP to induce maximal non-phosphorylating respiration likewise was without stimulatory effect in mitochondria exposed to calcium (Fig. 1A–B).

### Mitochondrial Calcium Retention

**Figure 2:**
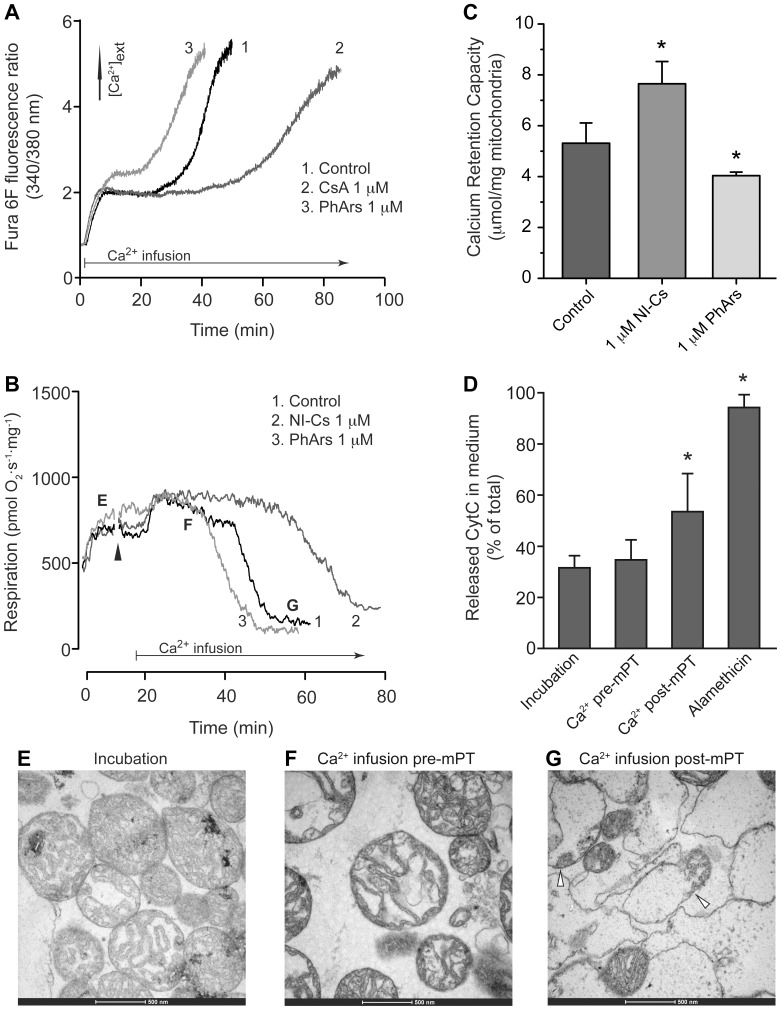
Effects of cyclophilin inhibition and oxidant on mitochondrial sensitivity to permeability transition during a continuous calcium infusion. **A)** Calcium retention capacity (CRC) was monitored by following fluorescence ratio of the extramitochondrial calcium-sensitive probe Fura 6F or **B)**, by following respiration changes. Experiments were performed in presence of the vicinal thiol reagent phenylarsine oxide (PhArs) under both experimental settings. The cyclophilin inhibitor cyclosporin A (CsA) was evaluated in calcium fluorescence experiments and the non-immunosuppressive CsA analog MeAla^3^EtVal^4^-cyclosporin/alisporivir (NI-Cs) in respiration experiments. Arrowheads indicate drug or vehicle addition. **C)** Calculations of CRC from experiments in panel B, calculated as amount of calcium retained from start of calcium infusion until start of the rapid phase of respiratory inhibition. **D)** Cytochrome *c* (CytC) release from mitochondria during CRC experiments in respiration chamber expressed as percent of total CytC present in mitochondria and supernatant. Samples were prepared before (incubation) and during calcium infusion (Ca^2+^ pre-mPT), after induction of respiratory inhibition (Ca^2+^ post-mPT) and following addition of the non-specific ionophore alamethicin. Values are means ± SD, n = 4–8 in panel C and n = 5 in panel D. *indicate p<0.05 using one-way ANOVA followed by Dunnett’s multiple comparison post hoc test. **E–G)** Electron micrographs prepared following sampling at time points indicated in panel B. Open arrowheads indicate examples of mitochondria with decreased cristae and expanded matrices.

Heart mitochondria exposed to a continuous calcium challenge buffered the infused calcium resulting in a steady state extramitochondrial Ca^2+^ concentration. The latter lasted until a threshold where retained calcium was released and extramitochondrial Ca^2+^ concentration was increased (Fig. 2A). The same type of experiment performed during measurement of oxygen consumption demonstrated a slight increase in respiration during calcium infusion followed by a rapid phase of respiratory inhibition, which defined the limit of mitochondrial CRC (Fig. 2B). The cyclophilin inhibitor NI-Cs (alisporivir) significantly increased CRC whereas the oxidant PhArs significantly decreased CRC (Fig. 2C). CsA was evaluated in the CRC assay using measurement of extramitochondrial Ca^2+^ concentration (Fig. 2A). Due to a high degree of variation in this set of experiments, the effect of CsA was only significant using paired analysis, *i.e.* when the experiments with CsA were compared to their respective controls (p = 0.034 using paired t-test, n = 4). CytC release from mitochondria was increased following the rapid phase of respiratory inhibition but not during the calcium infusion before (Fig. 2D). Electron micrographs indicated a dramatic alteration of morphological appearance following calcium-induced respiratory inhibition showing decreased cristae and expanded matrices (Fig. 2E–G).

### Reversible Swelling

**Figure 3:**
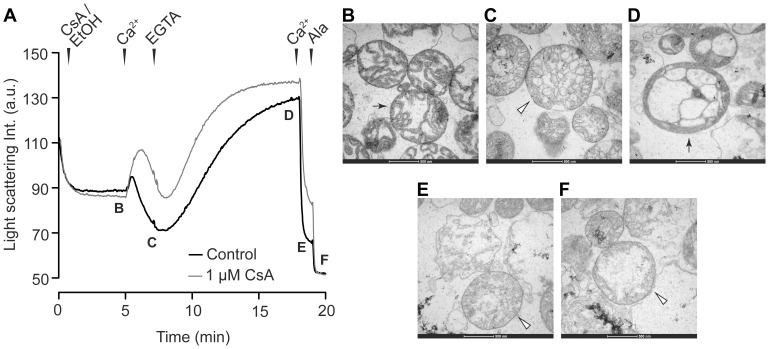
Light scattering and electron micrographs of mitochondria exposed to transient calcium insult. **A)** Light scattering of respiring mitochondria exposed to a transient calcium insult. Experiments were performed with 1 µM cyclosporin A (CsA, n = 2) or its vehicle ethanol (EtOH, Control, n = 5). A decrease in light scattering is attributed to swelling of mitochondria whereas an increase reflects reversal of swelling and accumulation of calcium-phosphate complexes [32], (a.u. = arbitrary units). Where indicated by arrowheads, mitochondria were exposed to 300 µM CaCl_2_ to induce swelling, 0.5 mM EGTA to chelate the CaCl_2_, a second addition of 400 µM CaCl_2_, and finally 10 µg/ml alamethicin. Electron micrographs were prepared at indicated time-points for control experiments as follows; **B)** during incubation, **C)** after initial CaCl_2_ addition, **D)** subsequent to EGTA, **E)** following second CaCl_2_ addition, and **F)** after alamethicin exposure. Arrows indicate mitochondria with condensed cristae. Open arrowheads indicate mitochondria with less condensed or disrupted cristae.

Respiring mitochondria exposed to a short-lasting bolus load of calcium demonstrated a decrease in light scattering, which was reversed following chelation of calcium by EGTA (Fig. 3A). Electron micrographs prepared during different time points in the experiment showed a transition from condensed to less condensed cristae following calcium exposure, consistent with the light scattering changes (Fig. 3B–C). Corresponding to the increase in light scattering following EGTA chelation of calcium, the mitochondrial cristae appeared hypercondensed (Fig. 3D). A second calcium exposure as well as subsequent addition of alamethicin induced decreased light scattering and appearance of disrupted cristae (Fig. 3E–F).

### Potency of Cyclophilin Inhibitors

**Figure 4:**
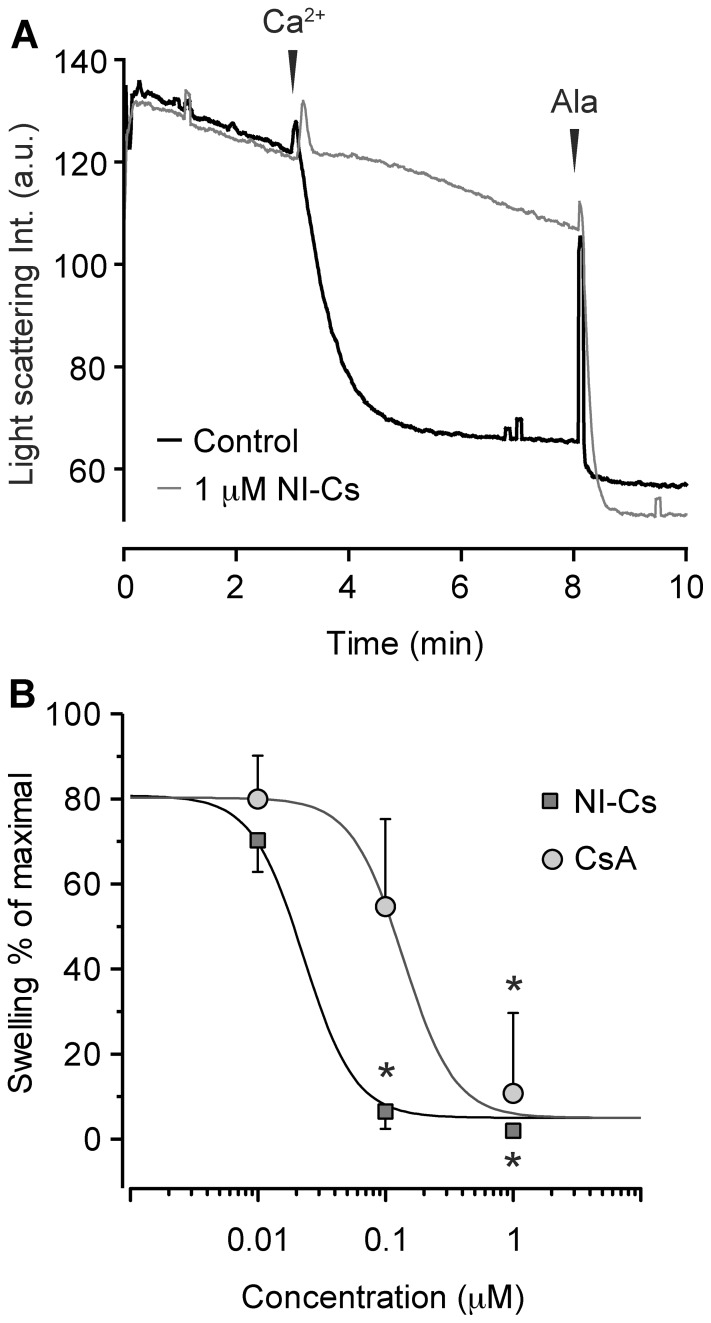
Inhibition of de-energized mitochondrial swelling by cyclophilin inhibitors. **A)** Mitochondrial swelling monitored by following light scattering, (a.u. = arbitrary units). Mitochondria were exposed to 300 µM Ca^2+^ and non-electrophoretic calcium uptake into mitochondria was mediated by the calcium ionophore A23187. Representative traces of experiments performed with 1 µM of the non-immunosuppressive cyclosporin MeAla^3^EtVal^4^-cyclosporin/alisporivir (NI-Cs) or with its vehicle ethanol (Control). The non-specific ionophore alamethicin (Ala) was added to induce a standardized maximal swelling response. **B)** Dose-response effect of swelling inhibition by cyclosporin A (CsA) and NI-Cs expressed as percent of that induced by alamethicin. Values are means ± SD with non-linear logarithmic dose-response curves. *indicate p<0.05 compared to control using one-way ANOVA followed by Dunnett’s multiple comparison post hoc test.

The potencies of the cyclophilin inhibitors CsA and NI-Cs to reduce calcium-induced swelling were compared under de-energized conditions (Fig. 4). Both compounds significantly inhibited swelling at 1 µM and NI-Cs also at 100 nM. The half maximal effective concentration (EC_50_) values were 138 nM for CsA and 23 nM for NI-Cs under the conditions used.

## Discussion

In the present study, we demonstrate that viable mitochondria from human cardiac tissue undergo calcium- and oxidant-sensitive mPT, which is morphologically reversible following a transient calcium insult. We also demonstrate that mPT activation in human heart mitochondria is inhibited by CypD ligands. Indications of mPT have previously been shown in human atrial myocytes and permeabilized muscle fibres [16]–[18]. Here, we used isolated mitochondria in order to more specifically evaluate mPT characteristics in the human heart.

Opening of the mPT pore allows passage of solutes with molecular weight below approximately 1500 Da [29], [30]. Respiratory uncoupling due to loss of proton motive force and respiratory inhibition due to loss of *e.g.* NAD(H) are more specific indications for mPT compared to *e.g.* changes in membrane potential probes in cell cultures. Although depolarization is a direct consequence of mPT pore opening, depolarization may not necessarily be caused by mPT. Further, a potential confounder when evaluating CsA effects and mitochondrial membrane potential is that membrane potential dyes such as TMRM as well as CsA are both substrates of the multidrug-resistance pump (MDR)/P-glycoprotein which may affect cellular loading and extrusion of the dyes [31]. The CRC assay is a sensitive and quantitative technique for assessing mitochondrial susceptibility to mPT [32]. The mPT pore is regulated by several endogenous factors such as redox status, adenine nucleotides, membrane potential and pH. Whether these factors will increase or decrease mPT activation in mitochondria actively buffering calcium will however also depend on their effects on intramitochondrial Ca^2+^ concentration *e.g.* by affecting calcium phosphate complex formation [25], [32], [33]. A potential pitfall when evaluating putative inhibitors of mPT using swelling or CRC assays is if the compound rather induces respiratory inhibition with reduced driving force for calcium uptake rather than direct modulation of mPT [20], [34]. By including simultaneous measurement of oxygen consumption and evaluation of the permeability of NAD(H), the specificity of *e.g.* the CRC assay for mPT is increased [25]. The findings of the present study with respiratory uncoupling and inhibition following calcium overload in combination with a PhArs-induced reduction and a CsA- and NI-Cs-induced increase in CRC demonstrate a specific activation of mPT in human heart mitochondria that is oxidant- and CypD-modulated. Whereas there are several cyclophilins present in the cell, there is no other target than CypD for the cyclosphilin inhibitors in mitochondria [14].

The increased permeability of mitochondria in vitro is reversible when mPT-inducing factors are removed [35], [36]. It has also been demonstrated that swelling is a morphologically reversible event if calcium is chelated following mPT activation [24], [37]. In the present study, we find evidence that a transient mPT can be induced in human heart mitochondria, as assayed by reversible light scattering decrease and reversible alteration of cristae appearance on electron micrographs, following a short-term calcium exposure. However, compared to the alteration following the transient calcium insult, the disruption of matrix cristae appeared more severe after a second calcium exposure (Fig. 3) or following mPT activation in the CRC experiments (Fig. 2), possibly due to the severity and duration of the calcium insult. A transient and reversible mPT has both been suggested to represent a physiological calcium release mechanism of mitochondria [38], and to underlie mitochondrial remodeling and a trigger of cell death following excitotoxicity [39], [40]. Transient mPT has also been proposed to mediate preconditioning in cardiac ischemia since cyclophilin inhibitors and genetic ablation of CypD were found to inhibit the protective effects of preconditioning [41], [42]. On the other hand, entrapment of 2-deoxyglucose was not detected following preconditioning only, whereas preconditioning inhibited entrapment of 2-deoxyglucose and improved mPT pore closure following ischemia [43]. Transient mPT thus seems to be activated during ischemia-reperfusion injury but it is more unclear whether it also plays a role in mediating ischemic preconditioning.

In order to further evaluate the pharmacological modulation of mPT in human heart mitochondria, the potencies of the CypD inhibitors CsA and NI-Cs were compared in a de-energized model of mitochondrial swelling. The absence of mitochondrial substrates and inhibition of the respiratory chain prevent the formation of a membrane potential and hence calcium uptake into the matrix, and by addition of a calcium ionophore, equilibration of calcium ions across the inner mitochondrial membrane is facilitated. Although less physiological and quantitative compared to the CRC assay, the de-energized model offers a specific and dose-dependent evaluation of regulation and pharmacological inhibition of the mPT pore components without interference from possible confounding effects on mitochondrial respiration, membrane potential and electrophoretic calcium uptake as well as calcium-phosphate complex formation [44]–[48]. The EC_50_ values of CsA and NI-Cs inhibition of mPT in de-energized mitochondria were somewhat higher in the present study (138 and 23 nM, respectively) compared to what we have previously demonstrated in rodent brain mitochondria using similar experimental conditions (49 and 5.5 nM, respectively) [49]. One possible explanation for the difference is the relative severity of the calcium insult which was higher in the present study. The relative difference in potency between CsA and NI-Cs was however similar.

The amount of calcium sequestered before induction of mPT in the CRC assay was somewhat higher in human heart mitochondria compared to what we have previously demonstrated in human brain and liver mitochondria using similar experimental conditions, 5.3±0.8 µmol/mg in heart versus 2.34±0.5 and 1.0±0.5 in human brain and liver mitochondria, respectively [19]. Rodent mitochondria display comparable tissue differences with a somewhat higher CRC in heart mitochondria compared to brain mitochondria and substantially higher CRC compared to liver mitochondria (unpublished observations and [20], [25], respectively). This indicates that under the conditions used, human heart mitochondria are more resistant to calcium-induced mPT than human brain and liver mitochondria. In contrast, studies exposing isolated mitochondria to bolus loads of calcium without exogenously added adenine nucleotides have demonstrated rodent heart mitochondria to be more sensitive to calcium-induced mPT compared to liver and brain mitochondria [50]. Such differences may be caused by different endogenous content of inhibitory factors of the mPT pore such as adenine nucleotides [51]. However, the comparison of mitochondria from different tissues has to be taken with some care as isolation of heart, liver and brain mitochondria require separate procedures, with possible different influence on both protective and sensitizing factors on mPT.

Several promising drugs in preclinical models have failed to translate into effective clinical use, which emphasizes the need to better validate the molecular targets chosen for drug discovery and development [52]. The mPT has been extensively characterized in animal models and may prove to be an important pathophysiological factor and pharmacological target in human cardiac disease. Historically, mitochondrial swelling has been noted since early studies of isolated mitochondria, and in order to attain well functioning mitochondria, a calcium chelator had to be present during the isolation process [53], [54]. The toxicity of calcium overload in heart has also since long been recognized [55]. Mitochondrial swelling was often considered an artifact until seminal studies in the late seventies established the mPT as a tightly regulated and reversible phenomenon. A pore formation as well as a physiological role was suggested [2], [29], [35], [44]. A decade later, it was proposed that mPT may have a major role in necrotic cell death associated with ischemia-reperfusion injury due to the associated changes in calcium, inorganic phosphate, adenine nucleotides and oxidative stress [36]. CsA was found to inhibit mPT [56], and CypD to be the mitochondrial target of CsA [45]. More recently, genetic ablation of CypD has been demonstrated to be cardioprotective [5], and CsA has shown promising results in a phase II clinical trial of myocardial infarction [12]. Identifying and characterizing the pharmacological target in human tissue is however an important translational step to improve the potential success of a compound targeting a disease mechanism. In the present study, we have demonstrated mPT in human heart mitochondria.

The majority of myocardial infarctions are located in the left ventricle but obstruction of the right coronary artery may also lead to involvement of the right ventricle and atrium. Similar to previous studies examining human heart mitochondria, the present study utilized atrial tissue as sampling from left ventricle is generally not feasible, and this is a limitation to the study. Further, the samples were obtained from a patient cohort with several cardiovascular disorders and medications, which could potentially influence the results of the present study, but these characteristics are however not atypical for patients undergoing ischemia-reperfusion injuries. Another limitation of the present study is the potential risk of morphological alterations induced during centrifugation and preparation of mitochondria for electron micrographs even though care was taken to rapidly process the mitochondria under cool conditions following the experimental procedures.

### Conclusion

Human heart mitochondria possess a CypD- and oxidant-regulated mPT similar to what has previously been characterized in non-human heart mitochondria as well as human brain and liver mitochondria [19], [20]. These findings support that inhibition of mPT may be a relevant pharmacological target in human cardiac disease and that it may underlie the beneficial effect of cyclosporin A in reperfusion injury.
